# Oleogels and Related Structured-Lipid Systems as Dual-Function Ingredients in Meat Products: Saturated Fat Replacement and Bioactive Delivery

**DOI:** 10.3390/foods15162803

**Published:** 2026-08-11

**Authors:** Bibiana Alves dos Santos, Néstor Sepúlveda, John Quiñones, Márcio Vargas-Ramella, Julián Andrés Gómez-Salazar, Paulo Cezar Bastianello Campagnol

**Affiliations:** 1 Departamento de Ciência dos Alimentos, Centro de Ciências Rurais, Universidade Federal de Santa Maria, Santa Maria 97105-900, RS, Brazil; paulo.campagnol@ufsm.br; 2 Centro de Tecnología e Innovación de la Carne (CTI-Carne), Universidad de La Frontera, Temuco 4780000, Chile; nestor.sepulveda@ufrontera.cl (N.S.); john.quinones@ufrontera.cl (J.Q.); 3 Facultad de Ciencias Agropecuarias y Medioambiente, Universidad de La Frontera, Temuco 4780000, Chile; 4 Centro de Educação Superior da Região Sul—CERES, Departamento de Ciências Biológicas, Universidade do Estado de Santa Catarina—UDESC, Laguna 88790-000, SC, Brazil; marcio.ramella@udesc.br; 5 Departamento de Alimentos, Division de Ciencias de la Vida, Campus Irapuato-Salamanca, Universidad de Guanajuato, Guanajuato 36500, Mexico; julian.gomez@ugto.mx

**Keywords:** oleogel, fat replacement, bioactive delivery, encapsulation, reformulated meat products, oxidative stability, meat matrix

## Abstract

Oleogels, in which liquid oils are structured into semi-solid matrices by oleogelators, have been widely investigated as animal fat replacers in meat products, where they lower saturated fatty acid content while helping preserve texture, structure, and emulsion stability. The reported gains are substantial: structuring PUFA-rich oils to replace pork backfat has raised the PUFA/SFA ratio around 3.6-fold and cut the n-6/n-3 ratio up to 23-fold in burgers, while cellulose- and wax-structured systems have reduced saturated fat from roughly 42% to 15% of total fat in patties. In parallel, oleogels and structurally related gels act as carriers for lipophilic bioactives such as carotenoids, curcumin, phytosterols, and omega-3 fatty acids, improving oxidative stability and modulating digestive release, with encapsulation efficiencies exceeding 90% in some systems. Whether one structured-lipid system can perform both roles inside a meat matrix remains largely untested: direct evidence for classical oleogels is confined to a few cases, most clearly curcumin-loaded beeswax oleogels in pork burgers, which retained antioxidant protection through chilled storage and cooking. This review integrates the fat-replacement and bioactive-delivery studies, separates classical oleogels from gelled emulsions, Pickering emulsions, and bigels, and stratifies studies by level of evidence. Because most current support comes from related systems, it is framed as a perspective that maps this evidence gradient and defines the experiments required to validate dual-function oleogels under realistic meat processing and storage conditions.

## 1. Introduction

Reducing the saturated fatty acid (SFA) content of processed meat products remains an important goal in applied meat science. Although the relationship between SFA intake and chronic disease risk depends on the overall dietary pattern and on the nutrients replacing SFA, reformulating meat products with oils richer in unsaturated fatty acids is widely explored as a strategy to improve their lipid profile. Among the technological approaches proposed for this purpose, oleogels have received particular attention over the past decade. An oleogel is formed when a liquid oil is physically entrapped within a three-dimensional network built by a structuring agent, or oleogelator, which imparts a solid- or semi-solid-like consistency to the oil without partial hydrogenation. Because the entrapped phase remains a liquid oil, oleogels can help mimic some functional properties of animal fat—such as plasticity, structure, and mouthfeel—while providing a fatty acid profile richer in unsaturated fatty acids [1,2].

The body of evidence supporting oleogels as fat replacers in meat products is substantial. Studies have applied oleogels structured with waxes, monoglycerides, phytosterols, hydroxypropyl methylcellulose (HPMC), ethylcellulose, and related structuring systems to burgers, frankfurter-type sausages, Bologna-type sausages, patties, liver pâté, dry-fermented sausages, and meat batters. In general, these studies report reductions in SFA content and improvements in nutritional lipid indices such as the PUFA/SFA and n-6/n-3 ratios. However, technological success is product-dependent. Changes in hardness, juiciness, cooking loss, lipid oxidation, color stability, sensory acceptance, and storage behavior have been reported depending on the oil, oleogelator, replacement level, and processing conditions used. Recent reviews have systematized this evidence in meat products [3,4], showing that oleogel-based fat replacement is a well-studied strategy, although its industrial adoption remains limited.

A second, largely independent line of research has examined oleogels not as fat replacers, but as delivery systems for lipophilic bioactive compounds. The oleogel network can reduce molecular mobility, restrict oxygen diffusion, and physically retain compounds within the structured lipid phase. For this reason, oleogels have been investigated as carriers for carotenoids, curcumin, tocopherols, phytosterols, essential oils, and omega-3 fatty acids, with several studies reporting improved oxidative stability or altered release during simulated digestion [5,6]. However, most of this evidence has been generated in isolated oleogels, model systems, bakery products, dairy systems, or matrices adjacent to the pharmaceutical industry. In non-meat systems, the underlying principle is already demonstrable: rapeseed-oil oleogels carrying green tea extract matched the oxidative stability of palm-fat biscuits while increasing unsaturated fatty acids and slowing lipid deterioration across fifteen weeks of storage [7], and comparable oxidative protection has been reported for candelilla-wax-structured model oil-in-water emulsions [8] and for olive oil emulsions used to reduce saturated fat in bakery products [9]. Direct evidence in meat products remains scarce, and this distinction is essential because meat matrices contain salt, water, proteins, heme pigments, iron, and endogenous pro-oxidants that may affect both bioactive stability and release.

The premise of this review is that these two functions may be mechanistically linked when oleogels are incorporated into meat products. The oleogelator selected to replace animal fat also determines the network’s density, porosity, oil-binding capacity, and thermal behavior, which could host any co-incorporated lipophilic bioactive compound. Thus, formulation decisions aimed at optimizing fat replacement may simultaneously affect bioactive retention, oxidative stability, and release. Reviews focused on meat products have mentioned bioactive delivery only briefly as a future opportunity, whereas reviews focused on bioactive delivery have rarely addressed the specific constraints of meat processing and storage. This review therefore brings together evidence from both research lines, distinguishing direct evidence in meat products from extrapolation from model or non-meat systems, and proposes a research agenda for evaluating oleogels as potential dual-function systems in processed meat products. Because unambiguous evidence for classical oleogels performing both roles inside a meat matrix is currently confined to isolated cases, this review deliberately treats structurally related systems (gelled emulsions, Pickering emulsions and bigels) as graded mechanistic support rather than as equivalent evidence, and its contribution is precisely to make that evidence hierarchy explicit and to specify what is needed to close it.

This review was prepared as a narrative and perspective-oriented synthesis. The literature was selected to represent two complementary bodies of evidence: studies using oleogels as animal fat replacers in meat products, and studies using oleogels or structurally related lipid gels as delivery systems for lipophilic bioactive compounds. Priority was given to studies that reported technological performance in meat products, bioactive retention or oxidative stability, sensory or storage outcomes, and mechanistic information related to oleogel structure. Recent reviews were also considered to identify consolidated findings and remaining gaps. Because the number of studies directly combining fat replacement with bioactive delivery in meat matrices remains limited, the review explicitly distinguishes between established evidence, hypotheses, and research opportunities. Thus, the main contribution of this review is not to claim that dual-function oleogels are already established in meat products, but to identify why their combined use is technically plausible, where the evidence remains insufficient, and which experimental approaches are needed to validate this strategy under realistic meat processing conditions.

## 2. Oleogel Structuring Systems

An oleogel forms when an oleogelator organizes into a three-dimensional network, immobilizing a liquid oil and converting it into a semi-solid material. For meat product reformulation, this network must perform several functions simultaneously: it must retain oil during mixing, chopping, stuffing, cooking, or drying; provide a texture compatible with the target product; remain stable during storage; and, if a bioactive compound is incorporated, protect or retain that compound within the lipid phase [10,11,12]. The main oleogel structuring routes differ in their mechanisms of gel formation, processing temperatures, network strengths, sensory impacts, and suitability for protecting bioactive compounds. These differences are summarized in Table 1.

Mechanistically, these routes differ primarily in how the oleogelator organizes the oil phase into a solid-like network. Crystal particle systems rely on the oleogelator itself crystallizing into needle-like, platelet-like, or lamellar structures that entangle and immobilize the surrounding oil [11]. Self-assembly systems, such as blends of γ-oryzanol and β-sitosterol or 12-hydroxystearic acid alone, instead nucleate within the oil phase and grow into fibrillar or helical structures stabilized by hydrogen bonding, van der Waals interactions, and hydrophobic forces [13]. Polymeric systems depend on a macromolecule capable of direct dispersion in oil. To date, ethylcellulose remains the principal polymer with this property which cross-links through hydrogen bonding into a continuous network once heated above its glass transition temperature and subsequently cooled, a step that typically requires temperatures above 100 °C and can itself promote oxidative degradation of the carrier oil and of any thermolabile bioactive compound present [11]. Indirect templated systems avoid this high-temperature step altogether by first building an aqueous emulsion, foam or hydrogel template with proteins, polysaccharides or cellulose ethers such as HPMC, and then removing the aqueous phase through drying, freeze-drying or supercritical carbon dioxide extraction, leaving a porous scaffold that is subsequently imbibed with liquid oil—a gentler route for oxidation-sensitive formulations, although the drying step introduces its own risk of oxidative aggregation at the oil–air interface [14,15].

Combining two or more oleogelators, such as wax and monoglyceride blends, sterol and monoglyceride blends, or polymeric systems reinforced with a secondary structurant, generally improves oil-binding capacity, thermal stability, and mechanical strength relative to mono-component systems; in ethylcellulose-wax blends, for example, adding as little as 1% wax allows a self-sustaining network to form at roughly half the ethylcellulose concentration (4% rather than the ~8% critical concentration required for EC alone) [11], consistent with the advantages summarised for multicomponent systems in Table 1. However, this advantage is not universal and depends on the target product, replacement level, processing conditions, and storage requirements. This point is particularly relevant for dual-function formulations, because the same structural properties that support fat replacement—network density, oil retention and thermal resistance—are also the properties most likely to govern bioactive retention and oxidative protection. Oleogelator type, concentration, and blend ratio should therefore be regarded not merely as texture-design variables, but as variables that may also shape the system’s potential to function as a bioactive-retention or delivery platform.

## 3. Saturated Fat Replacement in Meat Products

The application of oleogels as animal fat replacers has now been documented across essentially every category of processed meat product, and the accumulated evidence allows some general patterns to be drawn. In burgers, Gómez-Estaca et al. [10] structured a blend of olive, linseed, and fish oil with ethylcellulose or beeswax and used the resulting oleogels to fully replace pork backfat in low-fat, PUFA-enriched burgers, obtaining a 3.6-fold increase in the PUFA/SFA ratio and a 23-fold reduction in the n-6/n-3 ratio relative to the animal-fat control, with beeswax-structured burgers rated more favorably in sensory evaluation than their ethylcellulose counterparts. Moghtadaei et al. [16,17] explored sesame oil oleogels structured with beeswax or ethylcellulose as partial replacements for beef fat in beef burgers, reporting a 2-fold reduction in saturated fat content alongside improved textural attributes such as chewiness. However, oxidative stability tended to decline as the level of animal fat substitution increased. Lopes et al. [18] further evaluated pork-skin-structured olive oil oleogels in beef burgers, comparing virgin olive oil, stripped olive oil supplemented with olive-leaf extract, and stripped olive oil alone. Following cooking at 180 °C and 90 days of frozen storage, oxidation occurred only in burgers containing the antioxidant-depleted stripped oil, whereas both the virgin-oil and extract-supplemented formulations remained protected.

In frankfurter-type and other comminuted sausages, the evidence is similarly extensive. Barbut et al. [19] replaced up to 50% of pork backfat with a canola oil oleogel structured with ethylcellulose and sorbitan monostearate in breakfast sausages, finding that the addition of the surfactant was necessary to reproduce the objective hardness of the pork-fat control. Wolfer et al. [20] used soybean oil structured with rice bran wax to replace pork backfat in frankfurters, improving the fatty acid profile but also observing that oleogels prepared with the highest wax concentration required prolonged heating for complete dissolution, which accelerated lipid oxidation and introduced undesirable plastic or grassy sensory notes. Kouzounis et al. [21], whose work is discussed in more detail in the following section because of its direct relevance to the dual-function argument developed here, structured sunflower oil with a combination of monoglycerides and phytosterols to replace 50% of pork backfat in frankfurters. Da Silva et al. [12] adopted a markedly different structuring approach for Bologna-type sausages, combining pork skin, water, and high-oleic sunflower oil into a protein-based oleogel; they achieved a reduction in saturated fatty acids exceeding 30% at substitution levels of 50% or higher without significant sensory penalties. Igenbayev et al. [22] reported favorable sensory outcomes replacing pork fat with a beeswax/monoglyceride sunflower oil oleogel in semi-smoked sausages, particularly at lower substitution levels. More recently, Shao et al. [23] used an ethylcellulose–vegetable oil oleogel in Harbin red sausage, and Tanislav et al. [24] compared candelilla wax and glycerol monostearate in Bologna sausages. Both reported fatty acid improvements consistent with the earlier studies above, confirming that this line of research remains active and continues to test new oleogelator combinations within established product categories.

Patties, pâté and meat batters complete the picture. Oh et al. [25] structured canola oil with HPMC to replace beef tallow in meat patties, lowering the saturated fatty acid content from 42% to 15% of total fat while improving moisture retention and cooking loss. Martins et al. [26,27] applied sterol-based oleogels—linseed oil structured with a γ-oryzanol/β-sitosterol blend—to both pork patties and liver pâté, consistently improving the n-6/n-3 ratio and, in the case of the pâté, obtaining sensory scores for the 30% substitution level that remained close to those of the full-fat control. Agregán et al. [28] extended this sterol-based oleogel system in pork patties by co-incorporating a *Fucus vesiculosus* seaweed extract as a natural antioxidant, a study discussed further below given its relevance to the convergence between the two functions examined in this review. Gao et al. [29] reported that beeswax-structured rapeseed oil oleogel could replace all conventional fat in beef heart patties, reducing saturated fatty acids from 54 to 8 g per 100 g of fat and increasing polyunsaturated fatty acids more than threefold, although at the cost of reduced oxidative stability and softer texture during refrigerated storage. This outcome recurs across several of the studies cited here and is examined systematically in the section on technological trade-offs. In meat batters, Alejandre et al. [30] and Ferrer-González et al. [31] both found that oleogels performed more consistently than alternative structuring approaches, such as hydrogelled emulsions or seed pastes, in terms of microstructural uniformity and resistance to fat losses during cooking, reinforcing the broader conclusion that oleogel technology, across product categories, is capable of delivering a markedly improved lipid profile while imposing manageable, and in several cases negligible, sensory penalties.

Two further product categories complete the technological picture and have received comparatively little attention in earlier syntheses of this literature. In dry-fermented sausages, where prolonged exposure to salt, oxygen and dehydration during ripening imposes a markedly different stress profile than cooking, Pintado and Cofrades [32] compared a beeswax oleogel and a protein-stabilized emulsion gel, both prepared with an olive/chia oil blend, as pork backfat replacers in fuet-type sausages, reporting good oxidative and microbiological stability over 30 days of chilled storage regardless of the structuring method used. Zampouni et al. [33] combined an olive oil oleogel with partial NaCl-to-KCl substitution in fermented sausages, showing that fat and sodium reduction can be pursued simultaneously without disrupting fermentation or ripening, a combination directly relevant to reformulation strategies that target more than one nutrient at once. In poultry-based products, Tarté et al. [34] structured high-oleic and conventional soybean oil with rice bran wax to replace pork fat in mechanically separated chicken-based bologna sausage, improving stability and sensory attributes relative to the animal-fat control, although pork fat still provided a more intense color. These studies indicate that the technological feasibility of oleogel-based fat replacement extends beyond the burger-, frankfurter- and patty-type products that dominate the literature, into product categories with distinct processing stresses that are directly relevant to the kinetic questions raised later in this review. A related development concerns luncheon meat, a canned or vacuum-packed comminuted product with its own thermal sterilization profile: Ma et al. [35] constructed a linseed oil oleogel using soy protein isolate and highland barley β-glucan and used it to replace pork backfat in luncheon meat, reporting improved oil-binding capacity and restrained lipid oxidation relative to the unstructured oil. Finally, an emerging structuring category worth noting explicitly is the bigel, a biphasic system combining an oleogel and a hydrogel rather than a single oleogelator network. Siachou et al. [36] applied an olive oil bigel structured with monoglycerides, gelatin, and κ-carrageenan as a partial pork backfat replacer in fermented sausages, finding no adverse effect on water activity or lactic acid bacteria populations during fermentation. However, weight loss and TBARS values increased at the higher bigel inclusion level by the end of storage. Because bigels combine an aqueous and a lipid phase rather than structuring the oil phase alone, they fall outside the four routes described in Section 2. However, their growing presence in recent literature suggests that the boundary between oleogel-based and hybrid structuring strategies for meat products is becoming less distinct, a trend relevant to how the field is likely to develop over the next several years.

Taken together, this body of work establishes fat replacement as the primary and best-characterized function of oleogels in meat product reformulation. Nevertheless, improved fatty acid composition alone is not sufficient to define a successful reformulated meat product. Technological performance must also consider texture, cooking or processing yield, oxidative stability, color, sensory acceptance, storage behavior, and consumer perception. These parameters are particularly relevant when oleogels are also expected to carry bioactive compounds, because the same unsaturated oils that improve the nutritional lipid profile may also increase lipid oxidation susceptibility. The available meat product literature therefore provides a useful technological baseline, but not yet a complete demonstration of dual-function performance.

## 4. Oleogels as Delivery Systems for Bioactive Compounds

Outside the meat science domain, oleogels have been studied for more than a decade as encapsulation matrices for lipophilic bioactive compounds. The rationale is that the same network that immobilizes the oil phase may also reduce molecular mobility, limit oxygen diffusion, and protect sensitive compounds from degradation. Pinto et al. [37] described physical entrapment within crystalline or fibrillar networks, as well as more complex strategies in which the bioactive compound is first incorporated into an emulsion template before oleogel formation. Mehany et al. [5] further summarized applications of oleogels as carriers for polyphenols, omega-3 fatty acids, fat-soluble vitamins, essential oils, and related compounds across different food systems. However, this evidence should not be transferred directly to meat products without qualification, because meat matrices impose additional processing and chemical constraints.

Several compound-specific studies illustrate the magnitude of the protective effect that can be achieved. Li et al. [38] showed that surfactant-modified phytosterol oleogels conferred measurable protection to loaded curcumin, delaying its degradation relative to curcumin dissolved in unstructured oil, an effect attributed to reduced molecular mobility and restricted oxygen access within the gel network. In a complementary kinetic study, Keramat and Golmakani [6] found that structuring linseed oil into an oleogel altered both the antioxidant potency and the inhibitory mechanism of curcumin and its alkyl esters relative to the unstructured oil, indicating that the lipid system itself, and not only the compound, governs how effectively curcumin suppresses peroxidation. Working with β-carotene, Li et al. [39] proposed an ultrasound-assisted gelation method for candelilla wax and nut oil oleogels. They found that high-intensity ultrasound treatment reduced the release of β-carotene during simulated intestinal digestion, suggesting that processing parameters—not only oleogelator identity and concentration—can be tuned to modulate release kinetics. Chinnabutr and Chamchong [40] used ethylcellulose and beeswax to entrap bioactive compounds from gac (*Momordica cochinchinensis*) oil, reporting that the ethylcellulose-structured system preserved a significantly higher proportion of the original carotenoid and tocopherol content after three months of storage at room temperature than the unstructured oil control.

Although the majority of this evidence has been generated in model systems or in non-meat food matrices, the general principle of physical entrapment is relevant to meat products. However, the behavior of an oleogel network after incorporation into a sausage batter, burger, pâté, or dry-fermented meat product cannot be assumed to be identical to its behavior in isolation. Chopping, mixing, salt addition, protein extraction, cooking, smoking, drying, and refrigerated storage may disrupt the oleogel network or modify its interactions with the surrounding matrix. In addition, myofibrillar proteins, phosphates, salt, heme pigments, free iron, and endogenous pro-oxidants may affect both lipid oxidation and bioactive stability. Therefore, evidence from non-meat matrices should be treated as mechanistic support rather than direct proof of bioactive delivery in meat products.

## 5. Convergence of Both Functions in Meat Product Matrices

Direct evidence of oleogels performing both functions simultaneously within meat product matrices remains scarce. Therefore, the available studies should be interpreted as different levels of support for the dual-function concept rather than as definitive proof (Table 2). Kouzounis et al. [21], for example, did not evaluate bioactive delivery as its main objective. However, it is relevant because the oleogel was structured with phytosterols, compounds that may also have nutritional functionality. In this case, the structuring agent and the bioactive compound partly overlap. However, this should be considered an indirect example of convergence between structure and bioactivity, not evidence of encapsulation, controlled release, or bioaccessibility in the final meat product.

A more direct demonstration is provided by Gómez-Estaca et al. [41], who incorporated curcumin into ethylcellulose- and beeswax-structured oleogels prepared with a PUFA-rich oil blend (olive, linseed, and fish oil) and used them as full-fat replacers in low-fat pork burgers. Unlike most studies in this area, the burgers were evaluated not only immediately after formulation but also after simulated household storage and cooking, the two processing steps most relevant to the kinetic question raised later in this review. Curcumin measurably reduced lipid oxidation arising from both chilled storage and cooking. However, it imparted a yellow colour that lowered sensory acceptance regardless of the structuring method, and only the beeswax system proved technologically adequate—the ethylcellulose oleogels oxidized more extensively and scored lower. Even so, this study remains, to date, the clearest evidence that a bioactive compound co-incorporated into a fat-replacing oleogel can retain a protective function through the specific processing sequence characteristic of a real meat product, rather than only in the isolated oleogel or in a food system not subjected to comparable thermal treatment.

Agregán et al. [28] provide a very relevant example because a natural antioxidant extract from *Fucus vesiculosus* was incorporated into pork patties formulated with a linseed oil oleogel structured by γ-oryzanol and β-sitosterol. The extract was tested at different concentrations, and the 1000 mg/kg treatment was reported as the most effective in reducing lipid oxidation, whereas the 500 mg/kg treatment produced the highest odor scores after cooking. This study demonstrates the practical feasibility of combining an oleogel-based fat-replacement strategy with a bioactive antioxidant ingredient in a real meat matrix. Nevertheless, it remains unclear whether the extract was truly encapsulated within the oleogel network or co-formulated in the meat system. Therefore, the study should be interpreted as evidence of co-application rather than definitive evidence of controlled bioactive delivery. Lopes et al. [18] provided a particularly informative mechanistic comparison using beef burgers formulated with pork-skin-structured oleogels prepared from virgin olive oil, antioxidant-depleted stripped olive oil, or stripped olive oil supplemented with olive-leaf phenolic extract. After cooking at 180 °C and 90 days of frozen storage, oxidation developed only in the formulation containing stripped oil without added antioxidants, whereas both the virgin-oil and extract-supplemented systems remained protected. This stripped-versus-antioxidant contrast serves as a mechanistic control, indicating that the oxidative protection was attributable to endogenous or restored phenolic antioxidants rather than to oil structuring alone.

Yan and Zhang [42] evaluated peanut and soybean protein emulsion gels loaded with curcumin as partial or total substitutes for pork backfat in sausages. Although emulsion gels are structurally different from classical oleogels, this study is useful because it shows that a fat-structuring system can also carry a bioactive compound and remain functional after sausage processing. The reformulated sausages showed reduced cooking loss, improved textural attributes, and higher DPPH and ABTS radical-scavenging capacity than sausages prepared without curcumin. However, because the system was based on protein-stabilized emulsion gels rather than oleogels, it should be treated as a structurally related example and not as direct evidence of oleogel-mediated bioactive delivery.

Wang et al. [43] extended this type of evidence to a different bioactive compound and a structurally related delivery system, replacing fat in pork patties with a resveratrol-loaded Pickering emulsion stabilized by a myofibrillar protein–chitosan complex. As with Yan and Zhang [42], this system is not a classical oleogel. However, the outcome again supports the broader argument developed in this review: the reformulated patties showed improved antioxidant properties relative to the unloaded control, confirming that resveratrol, and not only curcumin, can remain functionally active when co-delivered with a fat-replacing structured lipid system in a real comminuted meat product.

A comparable, more recent example is provided by Alamer et al. [44], who enriched sunflower oil oleogels and emulgels with an onion peel phenolic extract and used both systems as fat replacers in beef burgers. The phenolic-enriched formulations showed improved oxidative stability relative to the non-enriched controls, and the study additionally evaluated texture and sensory attributes within the same experimental design, rather than treating these as separate lines of investigation. This combination of oxidative, textural, and sensory data within a single dual-function formulation is still uncommon in the literature. However, it is no longer absent, indicating that phenolic extracts beyond curcumin are technically compatible with oleogel-based fat replacement in comminuted meat products.

A related, though mechanistically distinct, contribution comes from Cofrades et al. [45], who compared an oleogel and a protein-stabilized gelled emulsion, both prepared with an olive/chia oil blend and loaded with curcumin, with respect to lipid hydrolysis and the in vitro bioaccessibility of curcumin and healthy fatty acids. The oil-structuring system significantly influenced both outcomes, and the authors explicitly proposed these systems as candidate fat replacers and bioactive carriers for future use in meat products. This study is valuable because it isolates the digestion-related behaviour of the structured lipid system itself, under standardized conditions, before the added complexity of a meat matrix is introduced; it does not, however, test bioaccessibility after the oleogel has been incorporated into an actual meat product and subjected to grinding, salting, and cooking. This distinction becomes central to the research agenda proposed in Section 7.

The longest direct test of dual-function stability comes from Noorolahi et al. [46], who incorporated pistachio green hull extract, a phenolic-rich antioxidant recovered from pistachio processing waste, into an ω-3-rich, carrageenan-structured linseed-oil gelled emulsion, which was used as a partial replacement for pork backfat in dry-fermented sausage. Properties were tracked throughout ripening and over two months of subsequent storage, a duration well beyond that reported in the other studies discussed in this section. The extract lowered TBARS values and preserved a higher proportion of ω-3 and polyunsaturated fatty acids relative to the antioxidant-free control, indicating that the protective effect of a co-incorporated bioactive compound can persist not only through cooking, as shown for burgers, but also through the markedly different combination of prolonged salt, oxygen, and dehydration exposure characteristic of dry-fermented products. As with the other structurally related systems discussed above, this gelled emulsion is not a classical oleogel, but the finding meaningfully extends the shelf-life evidence available for dual-function structured-lipid systems in meat products.

A more recent processing axis, largely absent from earlier syntheses of this literature, is three-dimensional food printing. Kang et al. [47] used a beeswax oleogel to reproduce the printing behaviour and lard-like texture of animal fat, optimizing wax concentration and infill level through a multi-response statistical design; the study addressed only technological performance, but it demonstrates that response-surface optimization of oleogel systems is already being extended to this processing context, a point directly relevant to the formulation-mapping gap discussed below. Cao et al. [48] went a step further, loading lycopene into a yeast-protein Pickering emulsion and using it as a fat replacer in 3D-printed meat products; encapsulation efficiency exceeded 90%, and lipid oxidation decreased as the level of fat replacement increased, indicating that the antioxidant remained functionally active through the printing process. As with the protein- and Pickering-emulsion-based systems discussed earlier in this section, this is not a classical oleogel, but it extends the dual-function evidence base to a processing technology that is likely to become more relevant as meat product manufacturing diversifies.

**Figure 1:**
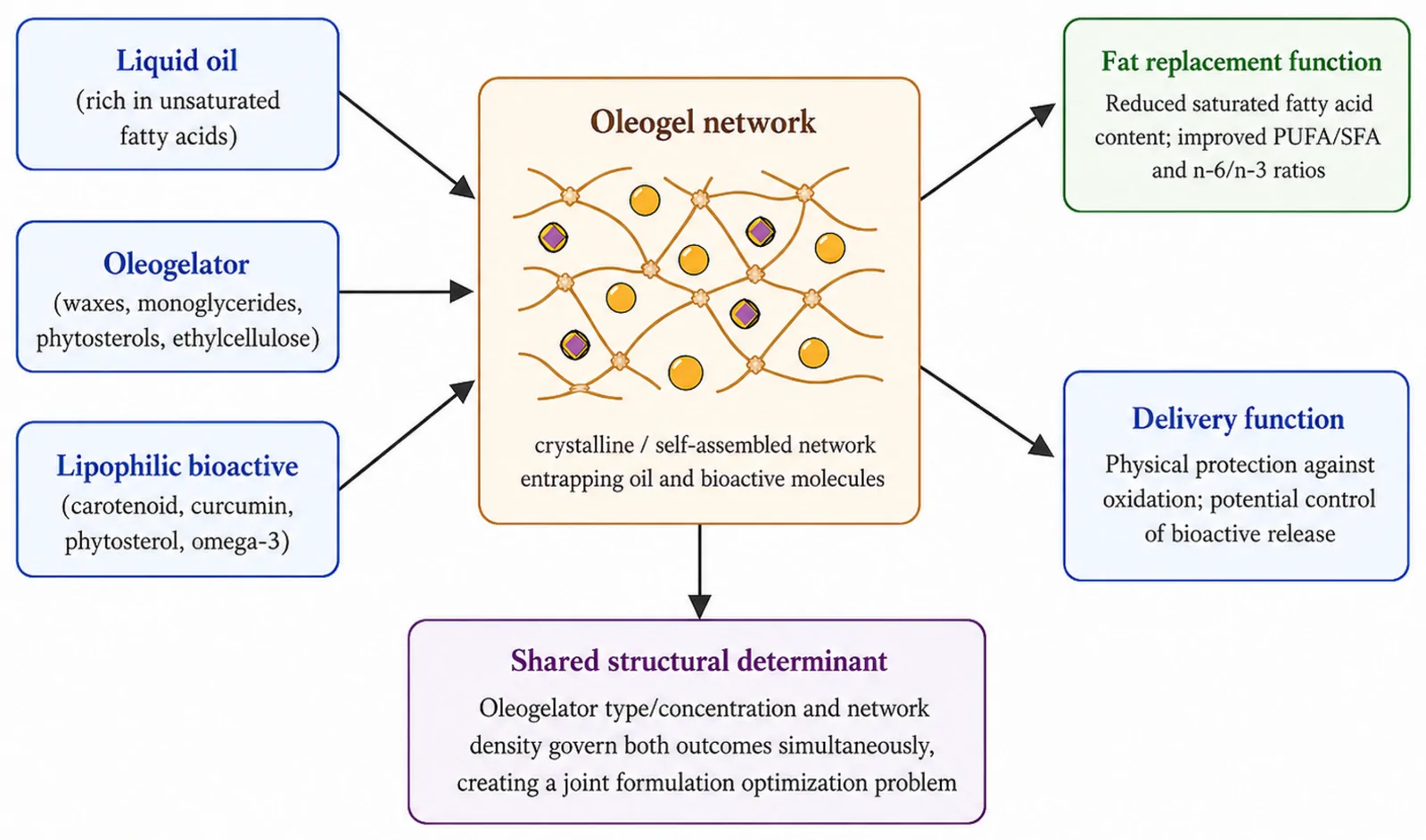
Conceptual scheme of the dual technological role of oleogel networks in processed meat products.

Taken together, these studies indicate that the convergence between fat replacement and bioactive delivery in meat matrices is technically plausible and, for structured-lipid systems more broadly, has been at least partially demonstrated across a range of products, oleogelators, bioactive compounds, and storage durations that are wider than earlier reviews in this area have acknowledged. For classical oleogels specifically, however, the direct evidence remains limited to a small number of cases—most clearly the curcumin-loaded oleogel burgers of Gómez-Estaca et al. [41]—with the remaining support coming from structurally related emulsion gels and Pickering emulsions. Current evidence spans at least four levels of inference: direct evidence for oleogels as fat replacers in meat products; direct evidence that a bioactive compound co-incorporated into a structured lipid system can retain a protective function through household storage and cooking [41], through processing accompanied by sensory evaluation [44], or through ripening and two months of storage in a dry-fermented product [46]; evidence that structurally related systems, such as protein-based emulsion gels and Pickering emulsions, can deliver curcumin or resveratrol with a measurable functional benefit in real meat products [42,43]; and evidence, so far restricted to isolated structured-lipid systems rather than finished meat products, on how the choice of structuring system affects the in vitro bioaccessibility of the encapsulated compound [45]. What remains largely unexplored is not whether this convergence is possible, but how oleogelator type, concentration, carrier oil composition, bioactive load, and meat processing conditions jointly determine its magnitude and consistency across product categories, and whether bioaccessibility is preserved once the same principles are tested in a finished, digested meat product rather than in the structured lipid system alone. Figure 1 summarizes this proposed shared structural basis.

**Table 2:** Evidence hierarchy for co-incorporating a bioactive compound into a fat-replacing structured-lipid system in, or intended for, meat products. Entries are ordered by strength of support for the dual-function concept in classical oleogels specifically (Levels A–D), so that the small number of direct oleogel studies and the larger body of related-system evidence are distinguished rather than pooled.

Study	Structuring System	Classical Oleogel	Bioactive	Meat Product	Processing/Storage Tested	Main Outcome	Evidence Level
Gómez-Estaca et al. [41]	Beeswax or ethylcellulose oleogel (olive/linseed/fish oil)	Yes	Curcumin	Low-fat pork burgers	Chilled storage + cooking	Curcumin reduced lipid oxidation from storage and cooking; beeswax adequate, ethylcellulose not; curcumin’s yellow colour lowered sensory acceptance	A—Direct
Agregán et al. [28]	Sterol-based oleogel (γ-oryzanol/β-sitosterol, linseed oil)	Yes	*Fucus vesiculosus* extract	Pork patties	Cooking + shelf life	1000 mg/kg most effective against lipid oxidation; 500 mg/kg best odour; encapsulation vs. co-formulation not resolved	B—Co-application
Alamer et al. [44]	Sunflower oil oleogel and emulgel	Partly	Onion-peel phenolic extract	Beef burgers	Oxidative + textural + sensory (same design)	Improved oxidative stability; combined oxidative/textural/sensory data in one dual-function formulation	B—Co-application
Lopes et al. [18]	Protein (pork-skin)-structured oleogel	Partly	Olive-leaf phenolic extract	Beef burgers	Virgin olive oil oleogel (HVOO); stripped olive oil + olive-leaf extract (HESOO); and stripped olive oil (HSOO)	After cooking at 180 °C and 90 days of frozen storage, only HSOO oxidised, while HVOO and HESOO prevented oxidation	B—Co-application
Kouzounis et al. [21]	Monoglyceride + phytosterol oleogel (sunflower oil)	Yes	Phytosterols (as structurant)	Frankfurters	Not applicable (no delivery test)	Structurant is itself nutritionally functional; no encapsulation, release, or bioaccessibility evaluated	Indirect—structure–bioactivity overlap
Yan & Zhang [42]	Peanut/soy protein emulsion gel	No	Curcumin	Sausages	Cooking	Reduced cooking loss, improved texture, higher DPPH/ABTS scavenging	C—Related system
Wang et al. [43]	Myofibrillar protein–chitosan Pickering emulsion	No	Resveratrol	Pork patties	Processing	Improved antioxidant properties vs. unloaded control	C—Related system
Cao et al. [48]	Yeast-protein Pickering emulsion	No	Lycopene	3D-printed meat	3D printing	Encapsulation efficiency > 90%; lipid oxidation decreased as fat replacement increased	C—Related system
Noorolahi et al. [46]	Carrageenan-structured linseed-oil gelled emulsion	No	Pistachio green-hull phenolic extract	Dry-fermented sausage	Ripening + 2 months storage (longest test)	Lowered TBARS; preserved ω-3 and PUFA vs. antioxidant-free control	C—Related system
Cofrades et al. [45]	Oleogel vs. protein gelled emulsion (olive/chia oil)	Compared	Curcumin	None (isolated structured lipid)	In vitro digestion (isolated)	Structuring system significantly affected lipid hydrolysis and in vitro bioaccessibility of curcumin and healthy fatty acids	D—Isolated system

Level A: classical oleogel + loaded bioactive tested in a meat product; B: oleogel + bioactive in meat, encapsulation unconfirmed; C: structurally related system (emulsion gel or Pickering emulsion) in meat; D: structured lipid tested in isolation. Kouzounis et al. [21] is classified separately because the structuring agent (phytosterol) is itself the functional compound, with no delivery evaluated.

## 6. Technological Trade-Offs and Limitations

The convergence between fat replacement and bioactive delivery is not without technical conflicts. The most important trade-off concerns oxidative stability. The same unsaturation that makes a carrier oil nutritionally attractive as an animal fat replacer may also increase susceptibility to lipid oxidation during chopping, cooking, smoking, drying, and refrigerated storage. This limitation has been reported in several oleogel-based meat reformulation studies [16,17,20,29], including undesirable sensory notes in high-wax frankfurters and reduced oxidative stability in patties and burgers formulated with unsaturated oil oleogels. When an antioxidant bioactive compound is co-incorporated, as in the study by Agregán et al. [28], it may partially compensate for this oxidative burden. However, the effectiveness of this strategy likely depends on bioactive concentration, oleogelator type, carrier oil composition, cooking temperature, oxygen exposure, storage time, and the pro-oxidant environment of the meat matrix. These variables have not yet been systematically evaluated across meat product categories.

A second point of tension concerns oleogelator concentration itself. Denser networks, obtained at higher oleogelator concentrations, may improve both the mechanical resemblance to animal fat and the retention of co-incorporated lipophilic compounds, since a tighter network can restrict molecular mobility and limit oxygen diffusion more effectively. However, the same increase in concentration frequently compromises sensory acceptability, either by producing an excessively firm or waxy texture, as noted for high rice bran wax concentrations by Wolfer et al. [20], or by introducing off-flavours associated with the structuring agent itself, particularly in ethylcellulose-based systems where sensory panels have repeatedly rated higher-concentration formulations less favourably than beeswax-based alternatives [4,10]. Optimizing bioactive retention, fat-replacement performance, and sensory acceptability simultaneously therefore occupies a narrower formulation space than optimizing any single objective in isolation, and the available literature—largely composed of single-objective optimization studies—provides limited guidance on where that space lies.

A third, more practical, constraint concerns processing temperature. Ethylcellulose-based oleogels, among the most widely used systems in the meat literature, typically require heating above 100 °C for complete polymer dissolution, a condition that can accelerate the oxidative degradation of thermolabile bioactive compounds such as carotenoids and certain phenolic classes before the product even reaches the meat matrix [11,39]. Indirect templated approaches that avoid the high-temperature step have been proposed as a gentler alternative. Still, their preparation introduces a drying step with its own oxidative risks at the oil–air interface, illustrating that no single oleogelation route is free of trade-offs when the goal is to preserve both structural performance and bioactive integrity. Finally, cost, labelling, and regulatory status should not be overlooked. Different oleogelators and bioactive compounds may have different regulatory classifications depending on jurisdiction, concentration, food category, and intended technological or nutritional function. Waxes, ethylcellulose, sterols, phytosterols, emulsifiers, plant extracts, and isolated bioactive compounds may not be equally accepted by regulators or consumers. In addition, the use of bioactive compounds at concentrations sufficient to produce measurable antioxidant, nutritional, or delivery effects may increase formulation cost. These issues are especially relevant for processed meat products, where reformulation strategies must remain compatible with sensory expectations, labelling constraints, industrial processing, and price competitiveness.

## 7. Research Gaps and Future Perspectives

Five research gaps emerge from the evidence assembled in this review (Table 2); several are already partly addressed, so each is stated below as the specific question that remains open. Kinetic persistence of a co-incorporated bioactive has been shown through household refrigerated storage and cooking in pork burgers [41]. Noorolahi et al. [46] demonstrated a comparable protective effect of a phenolic extract incorporated into a gelled emulsion during ripening and 2 months of storage in a dry-fermented sausage. What remains open is whether this kinetic behaviour generalizes further, across a wider set of product categories, bioactive classes, and, in the specific case of oleogels rather than structurally related gelled emulsions, whether the same protection holds for the thermal stress of cooked sausages and pâtés, which reach internal temperatures of 70–85 °C. Systematic comparisons across these processing regimes and across bioactive classes with thermal and oxidative sensitivities distinct from those of curcumin and phenolic acids, such as carotenoids or volatile phenolics, have not yet been reported.

The second gap concerns identifying formulation windows that jointly optimize saturated fat reduction, bioactive retention, oxidative stability, texture, and sensory acceptability. Response surface methodology has already been applied to lipid-structuring systems intended for meat products; Poyato et al. [49], for example, optimized a carrageenan-gelled oil-in-water emulsion for ω-3 delivery in Bologna-type sausages while tracking hardness and syneresis. However, this and similar studies optimize the structuring system around a single functional cargo, either the ω-3 oil itself or, in most oleogel studies, fat-replacement performance alone; none, to our knowledge, has varied oleogelator concentration and an added antioxidant or otherwise bioactive load simultaneously within a single multi-objective design applied to a finished meat product. Kang et al. [47] similarly applied multi-response optimization to a beeswax oleogel intended to reproduce the printing behaviour of animal fat, but, as before, around a purely technological objective, without an added bioactive load. This more specific formulation-mapping exercise, rather than the general application of response surface methodology to meat product reformulation, is what remains to be done.

The third gap concerns bioaccessibility. Cofrades et al. [45] have shown that the choice between an oleogel and a protein-stabilized gelled emulsion measurably affects the in vitro bioaccessibility of curcumin and healthy fatty acids. However, this comparison was performed on the structured lipid system in isolation, before its incorporation into a meat product. Whether the same relative differences hold once the oleogel has been ground, salted, emulsified with meat proteins, and cooked, and is then digested as part of a complete meat matrix rather than on its own, has not been tested. Myofibrillar proteins, phosphates, salt, heme iron, and the thermal load of meat processing are all plausible modifiers of bioactive release during digestion, and bioaccessibility data obtained from isolated structured-lipid systems, however informative, cannot be assumed to transfer directly to a finished meat product.

The fourth gap concerns the class of bioactive rather than its identity. The compounds tested so far—curcumin, seaweed and onion-peel phenolics, and resveratrol—are comparatively well characterized, with predictable colour, flavour, and antioxidant behaviour. Poorly defined phenolic-rich extracts from regional or underutilized plant materials behave less predictably: their pro- versus antioxidant balance in a heme-iron-rich meat system, their sensory contribution, and their regulatory status are largely unknown. They cannot be extrapolated from purified compounds. Whether such extracts can be co-incorporated into fat-replacing oleogels without compromising oxidative stability or sensory quality is therefore a genuinely open question.

The fifth gap concerns the application of shelf-life and sensory validation to specific commercial product formats. Combined oxidative, textural, and sensory evaluation within a single dual-function formulation has already been reported for burgers [41,44] and, for a gelled emulsion, across two months of ripening and storage in a dry-fermented sausage [46]. What has not been reported is an equivalent evaluation of classical oleogels specifically, over comparably long storage, in product categories such as sliced cooked sausages, where waxy mouthfeel, off-flavours, colour changes, and microbiological stability may differ from those in either burgers or dry-fermented sausages. This differs from the first gap, which concerns the mechanistic persistence of protection across processing regimes and bioactive classes; here, the question is whether that protection translates into acceptable, stable finished products of commercial relevance.

## 8. Conclusions

Oleogels have been extensively investigated as fat replacers in meat products, and a growing body of work has begun to test them, together with structurally related gelled emulsions, bigels, and Pickering emulsions, as delivery vehicles for lipophilic bioactive compounds within meat matrices. The central argument of this review is that these two functions are not independent: both are governed by the same structural variables, namely oleogelator type, oleogelator concentration, carrier oil composition, and the resulting network architecture, so that a formulation optimised for fat replacement simultaneously shapes bioactive retention, oxidative protection, and release.

Dual-function performance currently rests on preliminary and unevenly distributed evidence. The most direct support involves classical oleogels loaded with a model antioxidant and evaluated through refrigerated storage and cooking, whereas most remaining evidence derives from structurally related systems rather than from oleogels in the strict sense. The mechanistic case for coupling the two functions is therefore stronger than the direct oleogel evidence base, and the two should not be conflated. This evidence also remains fragmented, because individual studies differ in bioactive compound, structuring system, meat product, and processing or storage conditions, and were rarely designed to optimise fat replacement, bioactive retention, sensory quality, and shelf-life stability together.

The most consequential gap concerns classical oleogels evaluated in finished meat products under realistic processing, storage, and simulated digestion conditions, since bioaccessibility comparisons have so far been performed mainly on isolated structured-lipid systems. Consolidating this heterogeneous evidence into formulation windows that jointly optimise saturated fat reduction, bioactive retention, oxidative stability, sensory quality, and shelf life under industrially relevant conditions is the main priority emerging from this work. The question is no longer whether a dual-function role for oleogels is achievable, but how consistently, in which meat products, and under which processing and storage conditions it can be reproduced.

## Figures and Tables

**Table 1:** Comparison of oleogel structuring routes by typical oleogelators, technological advantages and limitations for meat product reformulation, and implications for bioactive delivery.

Structuring Route	Typical Oleogelators	Main Advantages for Meat Products	Main Limitations	Implications for Bioactive Delivery
Crystal particle systems	Beeswax, carnauba wax, candelilla wax, rice bran wax, monoglycerides	Efficient oil structuring at relatively low concentrations; useful for improving firmness and oil binding	Possible waxy mouthfeel; polymorphic instability; sensory defects at high concentrations	Dense crystalline networks may improve physical retention of lipophilic compounds, but high wax levels may reduce sensory acceptability
Self-assembled systems	γ-oryzanol/β-sitosterol, 12-hydroxystearic acid	Can form strong fibrillar networks; sterol-based systems may add nutritional relevance	Cost, regulatory status, and formulation complexity may limit application	Bioactive and structural roles may overlap when phytosterols act as oleogelators, but direct release data in meat matrices are limited
Polymeric systems	Ethylcellulose	Good mechanical tunability; useful for creating firm oleogels	Requires high processing temperatures; possible sensory limitations; high viscosity during preparation	High temperature may degrade thermolabile bioactives before incorporation into the meat matrix
Indirect templated systems	HPMC, methylcellulose, proteins, polysaccharides	Avoids direct high-temperature oil structuring; can generate porous scaffolds	Drying step increases complexity and cost; oxidation may occur during drying	Potentially useful for heat-sensitive bioactives, but stability after incorporation into meat products requires further study
Multicomponent systems	Wax/monoglyceride blends, sterol/monoglyceride blends, reinforced polymeric oleogels	Improved oil binding, thermal resistance, and texture modulation	More complex formulation; possible regulatory and sensory constraints	May provide better bioactive retention, but optimal conditions for simultaneous fat replacement and delivery remain unknown

## Data Availability

No new data were created or analyzed in this study. Data sharing is not applicable to this article.
